# Transforming growth factor-β1 induces fibrosis in rat meningeal mesothelial cells via the p38 signaling pathway

**DOI:** 10.3892/mmr.2022.12621

**Published:** 2022-01-27

**Authors:** Xue-Jing Yue, Yan Guo, Hai-Jie Yang, Zhi-Wei Feng, Tong Li, Yu-Ming Xu

Mol Med Rep 14: 1709-1713, 2016; DOI: 10.3892/mmr.2016.5411

Subsequently to the publication of this paper, while performing a careful re-examination of the scientific integrity of the data included in their publications, the authors have realized that they inadvertently used the incorrect western blotting images in Fig. 2B of this article, However, still having access to their original data, the authors were able to reassemble Fig. 2 correctly, and the corrected version of this figure is shown below.

Note that this error did not significantly affect the results or the conclusions reported in this paper, and all the authors agree to this Corrigendum. The authors thank the Editor of *Molecular Medicine Reports* for granting them the opportunity to publish this corrigendum, and apologize to the readership for any inconvenience caused.

## Figures and Tables

**Figure 2.**
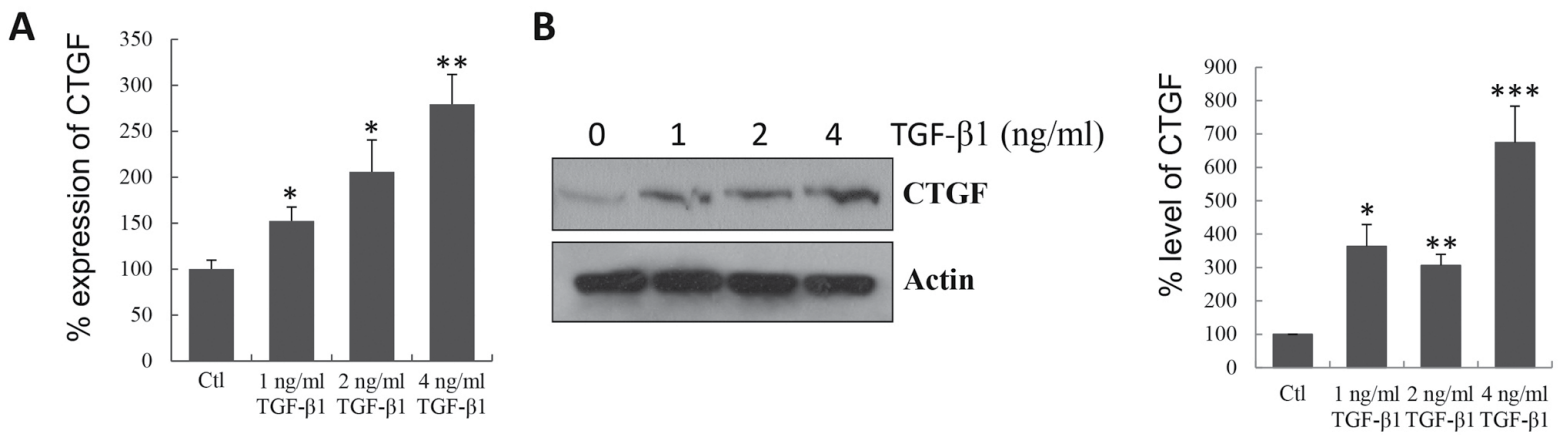
Fibrosis induction in MMCs by TGF-β1 *in vitro*. MMCs were treated with TGF-β1 at various concentrations for 2 days. (A) Quantitative polymerase chain reaction was conducted to determine the induction of CTGF mRNA. (B) Western blotting was performed to examine the induction of CTGF protein (upper panel), and the levels of CTGF protein are expressed as a percentage of the level measured in control cells (lower panel). *P<0.05, **P<0.01 and ***P<0.001 vs. control. MMCs, meningeal mesothelial cells; TGF-β1, transforming growth factor-β1; CTGF, connective tissue growth factor; Ctl, control.

